# Leaf Morphological Characters Can Be a Factor for Intra-Varietal Preference of Whitefly *Bemisia tabaci* (Hemiptera: Aleyrodidae) among Eggplant Varieties

**DOI:** 10.1371/journal.pone.0153880

**Published:** 2016-04-15

**Authors:** Abu Tayeb Mohammad Hasanuzzaman, Md. Nazrul Islam, Yi Zhang, Chen-Yang Zhang, Tong-Xian Liu

**Affiliations:** 1 State Key Laboratory of Crop Stress Biology for Arid Areas, Northwest A&F University, Yangling, Shaanxi, 712100, China; 2 Key Laboratory of Integrated Pest Management on the Loess Plateau of Ministry of Agriculture, Northwest A&F University, Yangling, Shaanxi, 712100, China; 3 Vertebrate Pest Division, Bangladesh Agricultural Research Institute, Joydebpur, Gazipur-1701, Bangladesh; 4 Agrochemical and Environmental Research Division, Institute of food and radiation Biology, Atomic Energy Research Establishment, Ganakbari, Savar, Dhaka, Bangladesh; University of Western Australia, AUSTRALIA

## Abstract

The sweetpotato whitefly, *Bemisia tabaci* (Hemiptera: Aleyrodidae) MEAM1, is considered a serious pest of horticultural and many other crops. While eggplant (*Solanum melongena*) is one of the most favored host plants, the whiteflies exhibit preferences among different varieties. We hypothesized that certain morphological leaf characteristics of different varieties, like leaf trichome density, trichome length, leaf lamina thickness and leaf color, may affect whitefly landing, feeding and oviposition. In this study, we investigated the variation in leaf morphological characters among selected eggplant varieties and evaluated the effect of these leaf characteristics in rendering eggplant varieties either susceptible or resistant to *B*. *tabaci*. We evaluated eight eggplant varieties in choice feeding tests, and we found that the varieties JinSheng Zilongchangqie (JSZ) and H149 were the highly preferred varieties with the highest numbers of whitefly adults and eggs. Significantly lower numbers of whitefly adult eggs were found on the resistant variety Tuo Lu Bamu (TLB). The varieties JinGuangbo Luqie (JGL), JinGuangbo Ziquanqie (JGZ), DaYang Ziguanqie (DYZ), QinXing Ziguanqie (QXZ), and QinXing Niuxinqie (QXN) were moderately favored by *B*. *tabaci*. Leaf trichome density, trichome length and leaf lamina thickness were positively correlated with numbers of whitefly adults and eggs. *B*. *tabaci* was less attracted to the leaves that reflect long and middle wavelength light (higher R and G values) than to the bright green leaves (medium G value), but the short wavelength light (higher B value) had no significant effect on whitefly preference. The degree of hue had a positive effect, and saturation and brightness had a negative effect on whitefly attraction.

## Introduction

The sweetpotato whitefly, *Bemisia tabaci* Gennadius MEAM1 (Hemiptera: Aleyrodidae), is one of the most widely distributed pests of vegetables, field crops, and ornamentals throughout the world [1, 2, 3]. *B*. *tabaci* was first recorded in China in the late 1940s [4], but it was not considered as a pest until the mid-1990s [5]. Since then, *B*. *tabaci* MEAM1 has swiftly displaced the indigenous population of this insect and rapidly spread through the entire country, and has caused serious damage and yield losses in many crops [6]. Researchers use many alternative names for *B*. *tabaci* but its formal revision with official description has not yet been determined [7]. In China, *B*. *tabaci* has a wide host range with a total of 361 plant species in 89 families including many field crops, vegetables and ornamentals in the families of Cruciferae, Compositae, Cucurbitaceae, Leguminosae and Solanaceae [8, 9, 10]. Shah and Liu [11] reported that considering developmental time and survival from egg to adult, *B*. *tabaci* performs better on eggplant than tomato, cucumber and pepper. *B*. *tabaci* is one of the most serious agricultural polyphagous pests worldwide, causing various physiological disorders in many crops and having the capacity to act as a vector to spread virus diseases through many crops of economic importance causing losses of millions of dollars per year [12, 13]. It causes damage through sucking plant sap from leaves, producing honeydew and ensuing sooty mold, and through transmission of plant viruses [14]. Potential losses caused by *B*. *tabaci* worldwide exceed $US300 million per year [15, 16].

Farmers generally use pesticides for the successful control of insect pests. However, many recent studies have shown that several routine pesticides including pyrethroids, acetamiprid, imidacloprid and nitenpyram have become ineffective in controlling *B*. *tabaci* due to its increasing level of resistance [17, 18, 19, 20]. Using varieties of host plants with behavioral resistance (antixenosis) can also be an effective, economic and ecofriendly method for controlling pests. Some of the recent work carried out on plant resistance to *B*. *tabaci* showed that this insect can beaffected by leaf morphological character such as leaf shape, hairiness and glandular trichomes [14]. In solanaceous crops like tomato, there was a positive correlation between total number of trichomes and the attractiveness to the adults as well as number of eggs laid [21, 22].

Trap cropping potentially reduces crop damage through integrated pest management (IPM) which is inexpensive and can simultaneously reduce the need for conventional pesticide applications [23, 24]. Trap cropping is a biologically based alternative control in which a highly preferred host plant is used to attract target insect pests away from a less-preferred main crop [25].

There have been few studies about the varietal preferences of *B*. *tabaci* in eggplant. In this study we investigated which were the most preferred for attracting whitefly and egg-laying out of eight eggplant varieties in relation to plant morphological characters. The detailed objectives of the study were to identify the most suitable variety that can be used by farmers as a trap crop for controlling whitefly, and, on the other hand, the most deterrent variety that can be cultivated by farmers as a tool of IPM without causing any detrimental effect to the environment. The variety with a high level of non-preference resistance can also be used by plant breeders as a source of resistance against *B*. *tabaci* in plant breeding programs.

## Materials and Methods

### Host plants

Eight eggplant (*Solanum melongena* L.) varieties, i.e. ‘JinGuangbo Luqie (JGL)’, ‘JinSheng Zilongchangqie (JSZ)’, ‘QinXing Niuxinqie (QXN)’, ‘QinXing Ziguanqie (QXZ)’, ‘DaYang Ziguanqie (DYZ)’, ‘JinGuangbo Ziquanqie (JGZ)’, ‘Tuo Lu Bamu (TLB)’ and H149 were used as host plants for the whiteflies. TLB, a wild variety that was considered as a control, and H149, a new eggplant line, were supplied by the Institute of Vegetables, Chongqing Academy of Agricultural Science (Chongqing, China). The remaining varieties were the commonly cultivated varieties that were purchased from a local vegetable seed market (Yangling, Shaanxi, China). All eggplants were seeded in plastic potting trays with a potting mix inside a growth chamber maintained at 24±1°C, 70±5% RH, a photoperiod of 16L: 8D h and a light intensity of 1400–1725 lux. The potting mix was prepared by mixing peat moss, perlite and vermiculite at a ratio of 5:1:1 by volume. For mass-rearing of whiteflies, the plant seedlings were transplanted individually to plastic pots (15 cm in diameter); whereas for bioassays, smaller plastic pots (10 cm in diameter) were used. The plants that had 5–6 true leaves were used for bioassays, and the second leaf from the top of the plant (the highest fully expanded leaf) was selected for all tests. All plants were watered as necessary and fertilized with a dry soluble fertilizer named “Harvest More 20-20-20+TE” at a rate of 1 g/L water at seven-day intervals.

### Whitefly culture

*Bemisia tabaci* was collected from the insectaries of the Key Laboratory of Applied Entomology at Northwest A&F University, Shaanxi, China, where it had been cultured previously on eggplant, *Solanum melongena* L. (Solanaceae) cv. ‘Zichangqie’. Only adult *B*. *tabaci* that was previously identified as MEAM1 [26] was used in this study. The whiteflies were cultured in large screen cages (60 × 60 × 60 cm) inside controlled insectaries with the environmental conditions of 25 ± 1°C, RH 60 ± 5%, photoperiod of 16L: 8D h and a light intensity of 1400–1725 lux. The same environmental conditions were maintained for conducting all tests. For bioassays and other tests, only newly emerged adult whiteflies (24 h to 48 h old) were used.

### Host selection and ovipositional choice

**Fig 1:**
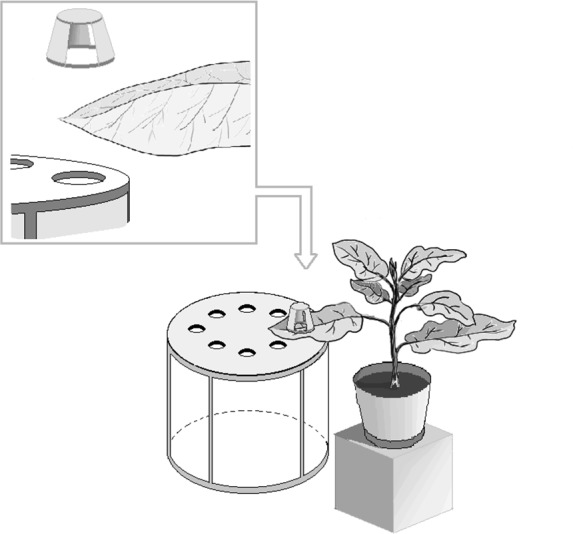
Diagrammatic view of host selection screen cage.

A barrel shaped test screen cage (40 cm high and 35 cm in diameter) was prepared to conduct the choice bioassay (Fig 1). The top of the cage was a non-transparent round plate (Acrylic sheet, 2 mm in thickness) in which eight circular holes (2.5 cm in diameter) were made to standardize the leaf area from each of the eight eggplant varieties. All holes were made at the same distance from the center and each other, and the hole to hole distance was 5.5 cm. To perform this test, the same numbers of leaves from the same aged plants were selected. Eight leaves, one from each tested eggplant variety, were placed randomly on a circular hole. The leaves were placed with the abaxial surface down on the holes to examine feeding performance. Therefore, only 2.5 cm in diameter of abaxial surface from each leaf were exposed. A plastic cup (3.5 × 3.5 cm) was placed inversely upon each of leaf on the hole to keep the leaves flat. Eighty adult whiteflies (40 male and female pairs), were released into the test screen cage over the eight exposed leaf surfaces. Numbers of whitefly adults on each leaf circle was counted after 12, 24 and 48 hours with the help of a mirror. After the termination of the test, the number of eggs laid by the whiteflies on each leaf portion was counted under a stereomicroscope. The completely randomized design was repeated so there were 10 replicates for each variety.

### No choice test

For the no choice oviposition test, a leaf clip on cage was used, made according to Mouttet et al. [27] with slight modifications. The clip cage was 3.5 cm in diameter and 4 cm in height and was made of a plastic cup with a metallic clip to hold the cage on abaxial leaf surface. There was a circular opening (2.5 cm in diameter) at the bottom of the cage which was covered with nylon mesh netting for ventilation, and a small hole (2–3 mm in diameter) was made at the side of cage to introduce adult whiteflies. The leaf clip-on cage was attached on the abaxial surface of a leaf of each of the eight eggplant varieties. One mated female (24–48 h old) collected from the whitefly colony was introduced into each clip-on cage, and the number of eggs laid on the abaxial surface of the leaf was counted three days after the introduction of the female. Four plants of each variety and two top fully expanded leaves were used. Each leaf was considered as one replication.

### Measurement of leaf trichome density and length

A square hole (0.5 cm × 0.5 cm) in a thin plastic transparent sheet was used to facilitate counting of trichomes. The punched plastic sheet was placed on the abaxial surface of the leaf lamina and the number of trichomes exposed through the hole was then counted using a stereomicroscope. Data were taken from the same position on the leaf in all treatments, and the same aged plants and the same numbers of leaves were maintained. Samples were taken from the intermediate position between the midrib and the leaf margin of the leaf lamina and between the top and the base of the leaf avoiding any dominant secondary veins. Ten replicates were conducted for each variety. In each replicate four values were taken from each leaf lamina, and a single meanwas used in statistical analyses.

To measure trichome length, only the most abundant star type trichomes were measured. Data was taken from eight leaves per variety; from each leaf, four star trichomes were picked off and placed on a glass slide. Under a stereomicroscope, a cover slip was placed on the trichome and pressed slightly to make the hairs straight. Then the length of trichome hairs was measured using a high definition compound microscope equipped with an internal micrometer. All hairs of each trichome were measured and compared among all eggplant varieties.

### Leaf thickness measurements

The same number of leaves was taken from the same aged varieties for measuring the thickness. Each leaf sample for measuring was taken from the intermediate position between the top and the base of the leaf and between the border and the midrib of the leaf lamina avoiding any dominant secondary veins. Each leaf sample was then placed into a potato block and a thin section cut with a sharp blade. The leaf sections were placed on a glass slide and examined with a high definition stereomicroscope ‘Discover V12 stereo ZEISS’ equipped with an Axiocam MRCS lens: 0.3XFWD 236 mm. Axiovision Rel 48 software was used for measuring the thickness. For each treatment, 10 sections (replicates) were measured. The mean value of leaf thickness for each replicate that included 5–6 values (taken from one leaf lamina) was used as a single value in statistical analysis.

### Leaf color measurement

For measuring leaf color, the same number of leaves from the same aged plants fo all eggplant varieties was taken. Leaf images were captured from the abaxial leaf surface in the same environmental conditions. The images were taken using a digital camera (Canon® EOS 5D Mark III, Canon, Japan) which was equipped with a high definition lens (Canon® Macro lens EF 100mm 1:2.8 L IS USM, Canon, Japan). To take the images, cold light source (KL 1500 LCD, Zeiss, German, set color temperature at 3200K) was used to maintain light supply, and the camera parameters were set in manual (shutter speed: 1/250; aperture: F4.0; ISO: 320; picture style: faithful 0, 0, 0, 0; white balance: color temp, 3,200 K, AF mode: manual focus; metering mode: center-weighted average). The images were recorded in RAW (CR2); 24-Patch color checker chart (Mennon, China) with standard colors on to captured images. The images were read and modified by the software Adobe Camera Raw and corrected by 24-patch color checker chart in Adobe DNG Profile Editor. Both RGB (Red-Green-Blue) and HSB (Hue-Saturation-Brightness) values were measured to describe the leaf color. Values of RGB channels were read and recorded by Adobe Photoshop CS6 and Microsoft Excel 2013.

### Data analysis

Statistical analyses were carried out using the IBM SPSS statistics version 19 (SPSS Inc., Chicago, IL, USA). The data were subjected to analysis of variance (ANOVA) to test the significance of variance among the eggplant varieties. The significant level was set at *P* < 0.05. Factorial design was applied to evaluate time of observations. The means were compared using the least significant different test (LSD). Morphological leaf characteristics were compared across varieties and correlations were used to identify a possible relationship between whitefly egg, adult density, and leaf characteristics of different varieties.

## Results

### Feeding and ovipositional choice

**Fig 2:**
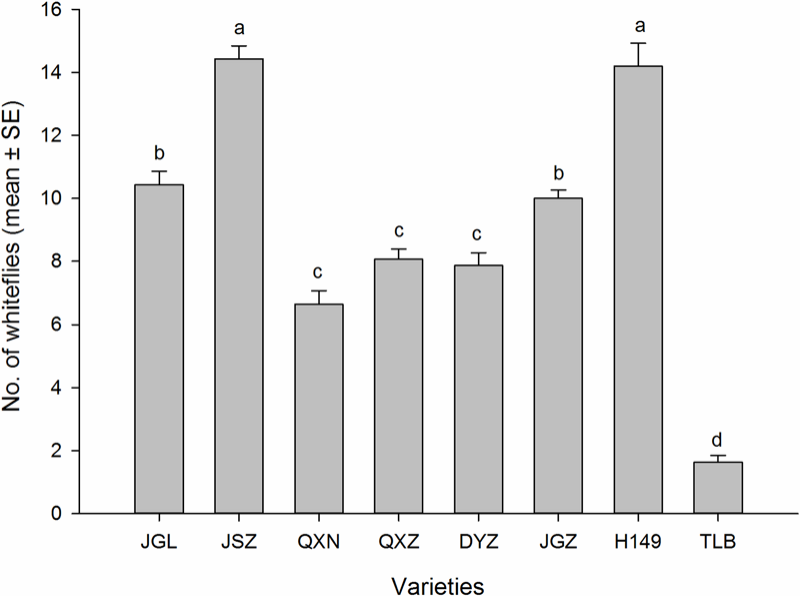
Numbers of *B*. *tabaci* adults (mean±SE) on leaf of the eight eggplant varieties in a choice bioassay. Different letters above the error bars indicate significant difference at *P* ≤ 0.05, LSD.

Adult distributions of the whitefly populations in the feeding choice experiment among various eggplant varieties are shown in Fig 2. The feeding choices of whitefly populations varied significantly among the eggplant varieties (*F*_7, 216_ = 90.27, *P* ≤ 0.001) The combined numbers of whiteflies in the three counts (after 12, 24 and 48 h of adult introduction) were significantly different among the varieties (*F*_7, 232_ = 94.87; *P* ≤ 0.001) (Fig 2). The highest numbers of adults were found on the varieties JSZ (14.43) and H149 (14.20), followed by the varieties JGL (10.43) and JGZ (10.00), then, the varieties QXZ, QXZ and DYZ, and the lowest was found on the variety TLB (1.63). At the end of the test the whitefly preference varied among the varieties which can be ranked from the highest to the lowest as: JSZ > H149 > JGL> JGZ > QXZ > DYZ > QXN > TLB.

**Fig 3:**
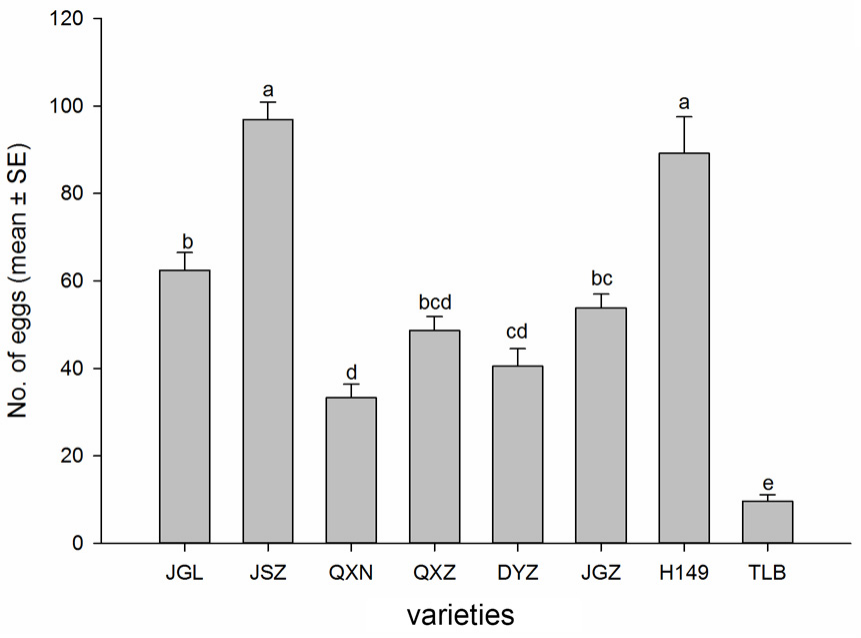
Number of *B*. *tabaci* eggs (mean±SE) on leaf of the eight eggplant varieties in a choice bioassay. Different letters above the error bars indicate significant difference at *P* ≤ 0.05, LSD.

The ovipositional preference of the whitefly adults was significantly different among all tested *S*. *melongena* varieties (*F*_7, 72_ = 43.99, *P* < 0.001) (Fig 3). The numbers of whitefly eggs recorded from different varieties ranged from 9.6 to 96.9 per leaf circle 48 hours after the adult whitefly release. The highest number of eggs was on the variety JSZ which was statistically similar to the variety H149, and the lowest number of eggs was on the variety TLB. The other varieties showed intermediate levels of ovipositional preference. The number of eggs had a highly significant and positive correlation with the number of whitefly adults (*r* = 0.857; *P* ≤ 0.001) landing on the leaf circles, indicating that the greater the number of whitefly adults, the more eggs were deposited.

### No choice oviposition

**Fig 4:**
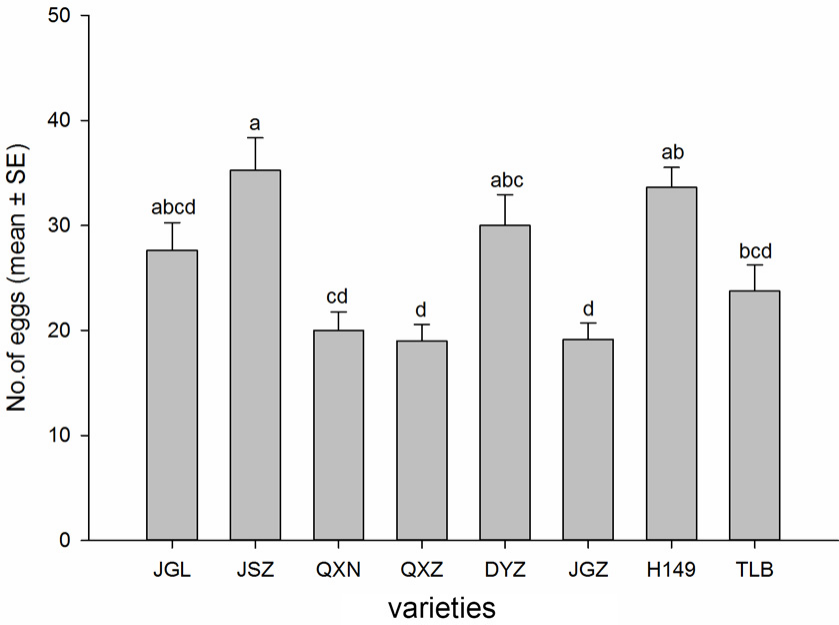
Number of *B*. *tabaci* eggs (mean±SE) on leaf of the eight eggplant varieties in a no-choice bioassay. Different letters above the error bars indicate significant difference at *P* ≤ 0.05, LSD.

The number of eggs laid in the no choice oviposition bioassay also differed significantly among the eggplant varieties (*F*_7, 56_ = 7.90, *P* < 0.001) (Fig 4). Whitefly egg numbers were highest on the variety JSZ (35.25 eggs per leaf circle) and lowest on the variety QXZ (19.00 eggs per leaf circle). Among all the test eggplant varieties, the variety JSZ recorded the highest number of eggs deposited, but was statistically similar to the varieties H149 (33.62), DYZ (30.00) and JGL (27.62). The whitefly adults laid fewer eggs on the varieties QXZ (19.00) and JGZ (19.12), and the lowest number of eggs was recorded on the varieties QXN (20.0) and TLB (23.75), which were not statistically different.

### Trichome density, trichome length and leaf lamina thickness

The variations in trichome density, trichome length and leaf thickness of eggplant varieties are presented in Table 1. Except for the variety TLB, which had both star type and glandular type trichomes, all varieties had only the star type (multi-radiate stellate non-glandular) trichome. The mean trichome density (star type) per 0.25 cm^2^ leaf area varied significantly among the varieties (*F*_7, 72_ = 252.30, *P* ≤ 0.001) where the variety H149 possessed the highest and the variety TLB had the lowest trichome density. The variety JSZ possessed the second highest density of trichomes, followed by the varieties JGL and JGZ, with densities similar to those of the varieties DYZ and QXN, and then followed by the variety QXZ.

**Table 1:** Mean number of leaf trichomes per 0.25 cm^2^ leaf area, trichome length (μm), and leaf thickness (μm) of selected eggplant varieties.

Varieties	Trichome No. (Mean ± SE)		Trichome length (Mean ± SE)	Leaf thickness (Mean ± SE)
	Star type	Glandular type		
JGL	78.50 ± 1.67 c	-	409.88 ± 6.82 b	352.75 ± 2.78 c
JSZ	104.30 ± 2.62 b	-	534.17 ± 10.24 a	438.15 ± 1.49 a
QXN	63.60 ± 1.74 e	-	241.68 ± 5.11 d	356.09 ± 2.17 c
QXZ	50.40 ± 2.01 f	-	282.27 ± 4.86 c	231.33 ± 2.47 e
DYZ	68.40 ± 1.17 de	-	239.53 ± 7.50 d	345.24 ± 2.60 c
JGZ	74.80 ± 2.59 cd	-	316.92 ± 9.39 c	374.94 ± 8.03 b
H149	145.30± 4.01 a	-	446.35 ± 12.58 b	325.65 ± 2.60 d
TLB	24.50 ± 0.75 g	122.9 ± 4.91	215.09 ± 6.05 d	164.21 ± 2.06 f
F value	*F*_7,72_ = 252.30	-	*F*_7,56_ = 196.43	*F*_7,72_ = 576.77
*P*	<0.001	-	<0.001	<0.001

Means within the same column followed by the same letter are not significantly different using Fisher’s LSD (α = 0.05).

Almost all the tested eggplant varieties possessed a multi-radiate stellate non-glandular trichome where one vertical hair and 7–8 horizontal hairs were present. The trichome length as well as hair length varied significantly (*F*_7,56_ = 196.43, *P* ≤ 0.001) among the varieties: the variety JSZ possessed the longest trichome and the variety H149 had the second longest, which was similar to that of the variety JGL. The varieties JGZ and QXZ had medium length of trichomes, whereas the variety TLB possessed the shortest trichome which was similar to those of the varieties DYZ and QXN.

The leaf lamina thickness of different eggplant varieties varied significantly (*F*_7, 72_ = 252.30, *P* ≤ 0.001). The variety JSZ recorded the largest, and the variety TLB recorded the smallest. The variety JGZ had the second highest, followed by the three varieties viz. QXN, JGL and DYZ, which had a similar thickness to the varieties H149 and QXZ.

### Leaf color

The variation in brightness of red (R), green (G) and blue (B) in leaves of different eggplant varieties are presented in Table 2, where significant differences were recorded (red: *F*_7, 32_ = 16.96, *P* < 0.001, green: *F*_7, 32_ = 21.40, *P* < 0.001, blue: *F*_7, 32_ = 3.53, *P* < 0.001). The highest *r* value, i.e., longest-wavelength reflection light, was found in the variety TLB, and the lowest *r* value was observed in the varieties JGZ and JGL where dark to bright scale was set as ‘0’ to ‘255’. The remaining varieties QXN, H149, QXZ, DYZ and JSZ showed a slightly brighter red color. Similarly, the highest G value was observed in the leaves of the variety TLB, and the lowest G value in the varieties JSZ and JGZ leaves. The leaves of the remaining varieties, i.e. QXN, H149, QXN, DYZ and JGL, displayed a moderately brighter color in G. Highest B value (short wave-length reflection light) was observed in the leaves of the varieties QXN, TLB and JSZ which were statistically similar to the varieties JGZ, DYZ, QXZ, and H149 whereas variety JGL showed the lowest B value. Considering all the color components, hue, saturation and brightness of leaf color also varied significantly (hue: *F*_7, 32_ = 11.59, *P* < 0.001; saturation: *F*_7, 32_ = 16.29, *P*<0.001; brightness: *F*_7, 32_ = 21.73, *P*<0.001) are presented in Table 2. The highest hue was found in the variety JGL leaves and the lowest was in the variety TLB leaf, and the remaining varieties showed moderate hues. On the other hand, the highest saturation was found in the variety TLB, followed by the varieties JGL and remaining varieties showed moderate saturation whereas the variety JSZ showed the lowest saturation. Considering all colors, the brightest color leaves were observed in the variety TLB, and the darkest leaves were observed in the variety JGZ, while the remaining varieties showed moderately bright leaves.

**Table 2:** Mean darkness/brightness of red (R), green (G) and blue (B) color, and hues, saturation and brightness of leaf lamina color of selected eggplant varieties.

varieties	Dark 0 ———————————— 255 Bright			Hue (°)	Saturation (%)	Brightness (%)
	R	G	B			
JGL	133.2 ± 0.73 c	148.6 ± 0.68 bc	69.4 ± 1.36 b	71.6 ± 0.24 a	53.4±0.87ab	58.4 ± 0.24 bcd
JSZ	133.8 ± 5.08 bc	145.2 ± 4.50 c	80.6 ± 3.68 a	70.6 ± 0.81ab	44.6±0.81d	57.0 ± 1.70 cd
QXN	145.0 ± 2.47 b	157.6 ± 1.83 b	83.0 ± 2.72 a	70.6 ± 0.24 ab	46.8±1.24cd	61.8 ± 0.66 b
QXZ	140.0 ± 1.45 bc	152.2 ± 1.91 bc	75.6 ± 1.25 ab	69.6 ± 0.24 ab	50.2±1.20bc	59.8 ± 0.80 bc
DYZ	138.6 ± 2.18 bc	151.4 ± 1.54 bc	78.0 ± 2.35 ab	70.4 ± 0.51 ab	48.2±1.24bcd	59.6 ± 0.68 bcd
JGZ	130.4 ± 1.33 c	142.6 ± 0.93 c	78.8 ± 2.71 ab	71.4 ± 0.24 ab	45.0±1.58cd	55.8 ± 0.37 d
H149	140.2 ± 1.59 bc	152.4 ± 1.57 bc	74.2 ± 1.83 ab	70.4 ± 0.24 ab	46.6±0.92cd	60.0 ± 0.55 bc
TLB	163.8 ± 2.84 a	175.6 ± 2.29 a	81.6 ± 2.08 a	67.0 ± 0.45 c	58.0±1.04a	68.8 ± 0.92 a
F value	*F*_7,32_ = 16.96	*F*_7,32_ = 21.40	*F*_7,32_ = 3.53	*F*_7,32_ = 11.59	*F*_7,32_ = 16.29	*F*_7,32_ = 21.73
P	<0.0001	<0.0001	0.006	<0.0001	<0.0001	<0.0001

Means within the same column followed by the same letter are not significantly different using Fisher’s LSD (α = 0.05).

### Correlations of leaf morphological characters and whitefly performance

Correlations between leaf characteristics and whitefly preferences are presented in Table 3. In the present study, a highly significant and positive correlation was recorded between the trichome density and the number of whitefly adults (*r* = 0.909; *P* ≤ 0.01) and eggs (*r* = 0.890; *P* ≤ 0.01). Similarly, leaf trichome length had a highly significant and positive correlation with the number of whitefly adults (*r* = 0.896; *P* ≤ 0.01) and eggs (*r* = 0.947; *P* ≤ 0.01). Moreover, leaf lamina thickness also showed a significant and positive correlation with the number of whitefly adults (*r* = 0.750; *P* ≤ 0.05) but not with eggs (*r* = 0.683; *P* = 0.062). Considering the combined effect of above three leaf characters, it was observed that these were significant and positively correlated with the number of adult whiteflies (*r* = 0.975; *P* ≤ 0.01) and eggs (*r* = 0.978; *P* ≤ 0.01), thus indicating that the higher the trichome density, trichome length and leaf lamina thickness, the more whitefly adults and eggs there would be. With regard to leaf color, the red had a significant and negative correlation with the number of whitefly adults (*r* = - 0.781; *P* ≤ 0.05) but not with eggs (*r* = - 0.693; *P* = 0.057). Moreover, green color intensity was negatively correlated with the whitefly adults (*r* = - 0.800; *P* ≤ 0.05) and eggs (*r* = -0.717; *P* ≤ 0.05), indicating that the higher the bright red and green color, the lower the number of adult whiteflies and less oviposition would be. However, no significant correlation was found between blue color intensity of the leaves and the whitefly adults (*r* = 0.310; *P* = 0.455) and eggs (*r* = 0.273; *P* = 0.513). Similarly, leaf color hue had no significant correlation between whitefly adults (*r* = 0.703; *P* = 0.052) and eggs (*r* = 0.591; *P* = 0.123). However, a significant and negative correlation was observed between the overall leaf color saturation and brightness with the whitefly adults (*r* = - 0.712, *P* ≤ 0.05; r = - 0.791, *P* ≤ 0.05).

**Table 3:** Correlation between morphological leaf characteristics of *Solanum melongena* and feeding and ovipositional preference of *Bemisia tabaci*.

Leaf characteristics	Correlation coefficient, *r (P* value)	
	Adults	Eggs
Trichome density (0.25 cm^2^ leaf area)	0.909 (0.002)	0.890 (0.003)
Trichome length (μm)	0.896 (0.003)	0.947 (0.000)
Leaf lamina thickness (μm)	0.750 (0.032)	0.683 (0.062)
Leaf lamina color:		
Red	-0.781 (0.032)	-0.693 (0.057)
Green	-0.800 (0.017)	-0.717 (0.045)
Blue	0.310 (0.455)	0.273 (0.513)
Hues (°)	0.703 (0.052)	0.591 (0.123)
Saturation (%)	-0.712 (0.048)	-0.632 (0.092)
Brightness (%)	-0.791 (0.019)	-0.708 (0.049)

## Discussion

In these experiments we measured adult whitefly preferences on different varieties of eggplant. This study was unconditional because the whiteflies had no previous experience with those varieties. In previous studies choice tests were conducted by placing the whole plant into a screen cage where the same leaf area was not maintained between varieties [28]. Leaf size can vary from plant to plant, which will affect the insect movement. Ozgur and Sckeroglu [29] reported that narrow leaved okra and super-okra cotton varieties conferred a higher degree of resistance to *B*. *tabaci* than broad-leaved cotton varieties. Furthermore, broad leaved varieties of cotton suffer more damage due to whiteflies compared with narrow leaved varieties [30]. In another study, leaf discs of the same size were cut and offered to insects for feeding and oviposition [11], but the mechanical wounding of the plants activated the oxylipin pathway and initiated the synthesis of green leaf volatile [31]. Green leaf volatiles can act as an attractant or a repellent for herbivores [32, 33, 34]. In case of the choice bio-assays, the above mentioned limitations can be minimized by using our test cages where the same leaf area can be provided to the insects without any damage to plant leaves.

In the present study, we found a significant variation in feeding and oviposition preferences by whitefly adults among the eggplant varieties. In the field this could affect the number of whiteflies on the different varieties. From our study, we found that the varieties H149 and JSZ were highly preferred while the variety TLB was less preferred for feeding and oviposition by the whitefly. Islam et. al. [35] found that eggplant variety ‘Baiyu’ was less preferred by *B*. *tabaci* than the other two varieties ‘Dafeng’ and ‘Beisite’ in China. We observed a positive relation between number of whitefly and number of eggs deposited in choice feeding and ovipositional tests. But in no choice ovipositional tests, some varieties like TLB which attracted fewer whiteflies had higher numbers of eggs. This is because the insect could not choose the host and they laid the eggs already developed in their reproductive system. So the relation between number of whiteflies and numbers of eggs laid were different in choice than in non-choice experiments.

Our data showed that morphological leaf characters like leaf trichome density, trichome length, leaf lamina thickness and leaf color played important roles in attraction and ovipositional choice of *B*. *tabaci*. We found that the highly preferred eggplant varieties possessed the highest densities and the longest trichomes, indicating that these two trichome characteristics may be a mechanism influencing the adult whitefly populations as well as oviposition. Trichome number, length and spatial arrangement appear to also influence the population density of whiteflies on different crops [36, 37]. Eggplant varieties having lower trichome densities recorded lower numbers of whitefly adults and eggs than other varieties. These results corroborate the findings of Ayyasamy and Baskaran [38], Singh et al. [39], and Soundararajan and Baskaran [40], who reported a negative correlation between trichome density in eggplant leaves and resistance to *B*. *tabaci*. Similar results have also been found by Rustamani et al. [41] in cotton, Silva et al. [42] in soybean, Oriani et al. [22] in tomato, and Shibuya et al. [43] in cucumber plant. Previous studies conducted on black gram [*Vigna mungo* (L.) Hepper] revealed a positive correlation between leaf trichome density and resistance to *B*. *tabaci* [44]. It has also been stated that the reason whitefly adults prefer to oviposit near trichomes is because of the selection pressure exerted by the natural enemies [36] or the improved microhabitat on the leaves [45]. The differences in feeding and ovipositional preference in whitefly adults may be due to the variation in other morphological factors, leaf chemical composition and green leaf volatiles produced by the plant species.

We also observed that only one eggplant variety possessed glandular trichomes, and it harbored the lowest whitefly population. This is a wild variety and considered as highly resistant against whiteflies (S. B. Tian, personal communication). The result is similar to the findings of Kennedy [46] and Shepherd [47] who reported that glandular trichomes function as an important source of chemical barriers for plant parasites. Similarly, Oriani et al. [22] and Oriani and Vendramim [21] observed a highly significant negative correlation in tomato plants between the density of glandular trichomes and attractiveness to and oviposition by whiteflies.

Our results demonstrated that the eggplant varieties possessing longer trichomes were more susceptible to *B*. *tabaci* than varieties possessing shorter trichomes. Similar results have also been reported by Singh et al. [39] who observed a positive correlation between whitefly population and trichome length in eggplant. Similarly, a negative correlation between trichome length and resistance to *B*. *tabaci* has been found in eggplant [40], beans [48], cotton [49], and black gram [44], where trichome length was positively correlated with whitefly population.

We also found that the less preferred eggplant varieties possessed thinner leaf laminae than the susceptible ones, which harbored a lower whitefly population and egg deposition than other varieties. The leaves with thinner laminae were less succulent and less preferred for feeding and oviposition by the whiteflies than the leaves with thicker laminae. This may be the possible reason for their not being attractive to the whiteflies. Similar results have been found in green gram [*Vigna radiate* (L.) R. Wilczek] [50], cotton [51], black gram [44] and cucumber [43], and leaf thickness in these crops was positively correlated with whitefly population. Although these results are not different to those of Ayyasamy and Baskaran [38] who reported that leaf thickness was correlated negatively with the occurrence of *B*. *tabaci* on eggplant, we found that some varieties had lower leaf thickness but their whitefly attractiveness was high, which could be caused by other leaf characters like trichome density and trichome length.

We observed,that the leaf lamina of the highly resistant eggplant varieties reflects long wavelength light than the susceptible varieties, and accordingly sheltered the lowest whitefly population. This result is supported by Frisbie et al. [52] who reported that red color of the leaf is an important feature of whitefly resistance in cotton. Our result is also supported by Elsey and Farnham [53] who observed less whitefly infestation in red leafed varieties of *Brassica oleracea*. Red foliage may serve as a true defensive cue, as it reliably indicates the plant’s low quality [54]. In this study, the eggplant varieties with the leaves that reflected more middle wave-length light (medium G value) were highly susceptible to *B*. *tabaci*. These results are similar to Petro and Redak [55] and Petro et al. [56] who showed that some light green leaf poinsettia varieties are more susceptible to *B*. *tabaci* than those with dark green leaves. Whiteflies are generally attracted to yellow or yellow-green [57, 58]. We found that leaves of some highly susceptible eggplant varieties reflect more short wavelength light. This finding is supported by Mound [58] who found that whiteflies were attracted to blue and ultraviolet lights. We also found that the leaves of some highly resistant varieties reflect more middle and short-wavelength lights, and this could be caused by their different chemical compositions and morphological characteristics, such as the presence of glandular trichomes. Further study is needed to confirm this result.

From this study we have shown that leaf morphological characteristics, like leaf trichome density, trichome length and leaf lamina thickness, had a positive effect on whitefly feeding and ovipositional preference. In case of leaf color brightness, the red and green had a negative effect on whitefly preference whereas the blue had no significant effect. Moreover, considering the whole color component of leaves among the eggplant varieties, the degree of hue had a positive effect, and the degree of saturation and brightness had a negative effect on whitefly preference. We suggested that in whitefly prone areas, eggplant varieties with thick and highly pubescent moderately brighter green colored leaves having long trichomes can be planted as trap crops to combat the whitefly infestation of main crops. Furthermore, the eggplant varieties with thin and less pubescent dark colored leaves having short trichomes are relatively tolerant to whiteflies, and can be planted to overcome the crop loss caused by whitefly. Further field trials are needed to evaluate the utility of using the most attractive varieties as trap crops, or the most tolerant or resistant varieties to reduce whitefly damage.

## References

[1] Wan FH, Zhang GF, Liu SS, Luo C, Chu D, Zhang YJ, et al (2009) Invasive mechanism and management strategy of *Bemisia tabaci* (Gennadius) biotype B: progress report of 973 program on invasive alien species in China. Sci China Ser C: Life Sci 25: 88–95. 10.1007/s11427-008-0135-4 19152088

[2] Dinsdale A, Cook L, Riginos C, Buckley YM, De Barro PJ (2010) Refined global analysis of *Bemisia tabaci* (Gennadius) (Hemiptera: Sternorrhyncha: Aleyrodoidea: Aleyrodidae) mitochondrial COI to identify species level genetic boundaries. Ann Entomol Soc Am 103: 196–208.

[3] De Barro PJ, Liu SS, Boykin LM, Dinsdale AB (2011) *Bemisia tabaci*: A statement of species status. Annu Rev Entomol 56: 1–19. 10.1146/annurev-ento-112408-085504 20690829

[4] Zhou Y (1949) A list of Aleyrodidae from China. Entomol Sin 3: 1–18.

[5] Luo C, Yao Y, Wang RJ, Yan FM, Hu DX (2002) The use of mitochondrial cytochrome oxidase mtCOI gene sequences for the identification of biotypes of *Bemisia tabaci* (Gennadius) in China. Acta Entomol Sin 45: 759–763.

[6] Liu SS, De Barro PJ, Xu J, Luan JB, Zang LS (2007) Asymmetric mating interactions drive widespread invasion and displacement in a whitefly. Science 318: 1769–1772. 17991828 10.1126/science.1149887

[7] Boykin L M. 2014 *Bemisia tabaci* nomenclature: lessons learned. Pest Manag Sci 70: 1454–1459. 10.1002/ps.3709 24338873

[8] Li SJ, Xue X, Ahmed MZ, Ren SX, Du YZ (2011) Host plants and natural enemies of *Bemisia tabaci* (Hemiptera: Aleyrodidae) in China. J Insect Sci 18: 101–120.

[9] Zhao L, Zhang R, Xiao Y, Cui Y, Huang W (2000) Tobacco whitefly (*Bemisia tabaci*), a new insect pest was founded on cotton in Xinjiang. Xinjiang J Agric Sci 1: 27–28.

[10] Lin K, Wu K, Wei H, Guo Y (2003) The effects of host plants on growth and development of *Bemisia tabaci* populations in China (Homoptera: Aleyrodidae). Acta Entomol Sin 23: 870–877.

[11] Shah MMR, Liu TX (2013) Feeding experience of *Bemisia tabaci* (Hemiptera: Aleyrodidae) affects their performance on different host plants. PLoS ONE 8: e77368 10.1371/journal.pone.0077368 24146985 PMC3795622

[12] Perring TM, Cooper AD, Rodriguez RJ, Farrar CA, Bellows TS Jr (1993) Identification of a whitefly species by genomic and behavioral studies. Science 259: 74–77. 8418497 10.1126/science.8418497

[13] Polston JE, Anderson PK (1999) Surgimientoy distribución de gemini virus transmit dospormoscablanca en tomate en el Hemisferio Occidental. Manejo Integrado Plagas 53: 24–42.

[14] Berlinger MJ (1986) Host plant resistance to *Bemisia tabaci*. Agric Ecosyst Environ 17: 69–82.

[15] De Barro PJ, Driver F (1997) Use of RAPD PCR to distinguish the B biotype from other biotypes of *Bemisia tabaci* (Gennadius) (Hemiptera: Aleyrodidae). Aust J Entomol 36:149–152.

[16] White J (1998) Silverleaf whitefly extends range. Calif Agric 52(2): 6–7.

[17] Wang ZY, Yao MD, Wu YD (2009) Cross-resistance, inheritance and biochemical mechanisms of imidacloprid resistance in B-biotype *Bemisia tabaci*. Pest Manag Sci 65: 1189–1194. 10.1002/ps.1808 19562662

[18] Byrne FJ, Oetting RD, Bethke JA, Green C, Chamberlin J (2010) Understanding the dynamics of neonicotinoid activity in the management of *Bemisia tabaci* whiteflies on poinsettias. Crop Prot 29: 260–266.

[19] Feng YT, Wu QJ, Wang SL, Chang XL, Xie W, Xu BY, et al (2010) Cross-resistance study and biochemical mechanisms of thiamethoxam resistance in B biotype *Bemisia tabaci* (Hemiptera: Aleyrodidae). Pest Manag Sci 66: 313–318. 10.1002/ps.1877 19937914

[20] Schuster DJ, Mann RS, Toapanta M, Cordero R, Thompson S, Cyman S, et al (2010) Monitoring neonicotinoid resistance in biotype B of *Bemisia tabaci* in Florida. Pest Manag Sci 66: 186–195. 10.1002/ps.1853 19790225

[21] Oriani GMA, Vendramim JD (2010) Influence of trichomes on attractiveness and ovipositional preference of *Bemisia tabaci* (Genn.) B biotype (Hemiptera: Aleyrodidae) on tomato genotypes. Neotrop Entomol 39: 1002–1007. 21271071 10.1590/s1519-566x2010000600024

[22] Oriani GMA, Vendramim JD, Vasconcelos CJ (2011) Biology of *Bemisia tabaci* (Genn.) B biotype (Hemiptera: Aleyrodidae) on tomato genotypes. Sci Agric 68: 37–41.

[23] Cavanagh A, Hazzard R, Adler LS, Boucher J (2009) Using trap crops for control of *Acalymma vittatum* (Coleoptera: Chrysomelidae) reduces insecticide use in butternut squash. J Econ Entomol 102: 1101–1107. 19610425 10.1603/029.102.0331

[24] Lu YH, Wu KM, Wyckhuys KAG, Guo YY (2009) Potential of mungbean, *Vigna radiatus* as a trap crop for managing *Apolygus lucorum* (Hemiptera: Miridae) on Bt cotton. Crop Prot 28: 77–81.

[25] Shelton AM, Badenes-Perez FR (2006) Concepts and applications of trap cropping in pest management. Ann Rev Entomol 51: 285–308. 16332213 10.1146/annurev.ento.51.110104.150959

[26] Frohlich DR, Torres JI, Bedford ID, Markham PG, Brown JK (1999) A phylogeographical analysis of *Bemisia tabaci* species complex based on mitochondrial DNA markers. Mol Ecol 8: 1683–1691. 10583831 10.1046/j.1365-294x.1999.00754.x

[27] Mouttet R, Bearez P, Thomas C, Desneux N (2011) Phytophagous arthropods and a pathogen sharing a host plant: Evidence for indirect plant-mediated interactions. PLoS ONE 6: e18840 10.1371/journal.pone.0018840 21611161 PMC3097179

[28] Lee DH, Nyrop JP, Sanderson JP (2009) Attraction of *Trialeurodes vaporariorum* and *Bemisia argentifolii* to eggplant, and its potential as a trap crop for whitefly management on greenhouse poinsettia. Entomol Expt Appl 133: 105–116.

[29] Ozgur AF, Sckeroglu E (1986) Population development of *Bemisia tabaci* (Homoptera: Aleyrodidae) on various cotton varieties in Cukurova, Turkey. Agric Ecosyst Environ 17: 83–88.

[30] Misra CS, Lamba KS (1929) The cotton whitefly (*Bemisia gossypiperda* n. sp.). Bull Agril Res Pusa 196: 7.

[31] Halitschke R, Stenberg JA, Kessler D, Kessler A, Baldwin IT (2008) Shared signals–‘alarm calls’ from plants increase appearance to herbivores and their enemies in nature. Ecol Lett 11: 24–34. 17961175 10.1111/j.1461-0248.2007.01123.x

[32] Dicke M, Baldwin IT (2010) The evolutionary context for herbivore-induced plant volatiles: beyond the ‘cry for help’. Trends Plant Sci 15: 167–175. 10.1016/j.tplants.2009.12.002 20047849

[33] Mumm R, Dicke M (2010) Variation in natural plant products and the attraction of bodyguards for indirect plant defense. Can J Zool 88: 628–667.

[34] Unsicker SB, Kunert G, Gershenzon J (2009) Protective perfumes: the role of vegetative volatiles in plant defense against herbivores. Curr Opin Plant Biol 12: 479–485. 10.1016/j.pbi.2009.04.001 19467919

[35] Islam MT, Qiu BL, Ren SX (2010) Host preference and influence of the sweet potato whitefly, *Bemisia tabaci* (Homoptera: Aleyrodidae) on eggplant (*Solanum melongena* L.). Acta Agric Scan, Section B–Soil Plant Sci 60: 320–325.

[36] Heinz KM, Zalom FG (1995) Variation in trichome based resistance to *Bemisia argentifolii* (Homoptera: Aleyrodidae) oviposition on tomato. J Econ Entomol 88: 1494–1502.

[37] Kishaba AN, Castle S, McCreight JD, Desjardins PR (1992) Resistance of white-flowered gourd to sweet potato whitefly. Hort Sci 27: 1217–1221.

[38] Ayyasamy R, Baskaran P (2005) Influence of certain leaf characters of brinjal accessions with incidence of *Bemisia tabaci*. J Food Agric Environ 3: 333–334.

[39] Singh D, Jaglan RS, Singh R (2002) Leaf morphological characteristics of brinjal in relation to whitefly incidence. Haryana J Hort Sci 31: 289–291.

[40] Soundararajan RP, Baskaran P (2001) Mechanisms of resistance in brinjal (*Solanum melongena* L.) to whitefly *Bemisia tabaci* (Gennadius). Madras Agric J 88: 657–659.

[41] Rustamani MA, Khatri I, Leghari MH, Sultana R, Mandokhail AS (2014) Trichomes of cotton leaf as an aspect of resistance to sucking insect pests. Sindh Univ Res J (Sci Ser) 46: 351–356.

[42] Silva GFD, Paulo J, Baldin L, Luiz E, Souza SD, Lourencao EY, et al (2012) Assessing *Bemisia tabaci* (Genn.) biotype b resistance in soybean genotypes: antixenosis and antibiosis. Chilean J Agric Res 72: 516–522.

[43] Shibuya T, Hirai N, Sakamoto Y, Komuro J (2009) Effects of morphological characteristics of *Cucumis sativus* seedlings grown at different vapor pressure deficits on initial colonization of *Bemisia tabaci* (Hemiptera: Aleyrodidae). J Econ Entomol 102: 2265–2267. 20069856 10.1603/029.102.0631

[44] Taggar GK, Gill RS (2012) Preference of whitefly, *Bemisia tabaci*, towards black gram genotypes: Role of morphological leaf characteristics. Phytoparasitica 40: 461–474. 10.1007/s12600-012-0247-z

[45] Chu CC, Henneberry T, Cohen AC (1995) *Bemisia argentifolii* (Homoptera: Aleyrodidae): host preference and factors affecting oviposition and feeding site preference. Envirol Entomol 24: 354–360.

[46] Kennedy GG (2003) Tomato, pests, parasitoids, and predators: Tritrophic interactions involving the genus *Lycopersicon*. Annu Rev Entomol 48: 51–72. 12194909 10.1146/annurev.ento.48.091801.112733

[47] Shepherd RW, Bass WT, Houtz RL, Wagner GJ (2005) Phylloplanins of tobacco are defensive proteins deployed on aerial surfaces by short glandular trichomes. Plant Cell, 17, 1851–1861. 15894716 10.1105/tpc.105.031559 PMC1143082

[48] Oriani GMA, Vendramim JD, Brunherotto R (2005) Influence of trichomes on ovipositional preference of *Bemisia tabaci* (Genn.) biotype B (Hemiptera: Aleyrodidae) for bean genotypes. Neotrop Entomol 34: 97–103.

[49] Acharya VS, Bhargava MC (2008) Morphological basis of resistance in cotton (*Gossypium hirsutum*) against whitefly (*Bemisia tabaci*). Indian J Agril Sci 78: 818–820.

[50] Lakshminarayan S, Singh PS, Mishra DS (2008) Relationship between whitefly population, YMV disease and morphological parameters of green gram germplasm. Environ Ecol 26: 978–982.

[51] Butter NS, Vir BK (1989) Morphological basis of resistance in cotton to the whitefly *Bemisia tabaci*. Phytoparasitica 17: 251–561.

[52] Frisbie RE, Reynolds HT, Adkisson PL, Smith RF (1994) “Cotton insect pest management”, in Introduction to Insect Pest Management, 3rd ed. (Eds.): Metcalf R. L. and Luckman W. H. (John Wiley & Sons, Inc New York): 421–468.

[53] Elsey KD, Farnham MW (1994) Response of *Brassica oleracea* L. to *Bemisia tabaci* (Gennadius). Hort Science 29: 814–817.

[54] Maskato Y, Talal S, Keasarand T, Gefen E (2014) Red foliage color reliably indicates low host quality and increased metabolic load for development of an herbivorous insect. Arthropod Plant Interac 8: 285–292.

[55] Petro LO, Redak RA (2000) Host plant preference and performance of *Bemisia argentifolii* (Homoptera: Aleyrodidae) on poinsettia (*Euphorbia pulchirrima*) in relation to variety. US Dept Agric, Agric Res Serv July 2000.

[56] Petro L, Redak R, Bethke J, Perring TM (2002) Preference and performance of silverleaf whitefly on selected poinsettia varieties. US Dept Agric, Agric Res Serv June 2002.

[57] Berlinger MJ (1980) A yellow sticky trap for whiteflies: *Trialeurodes vaporariorum* and *Bemisia tabaci* (Aleyrodidae). Entomol Expt Appl 27: 98–102.

[58] Mound LA (1962) Studies on the olfaction and colour sensitivity of *Bemisia tabaci* (Germ.) (Homoptera, Aleyrodidae). Entomol Expt Appl 5: 99–104.

